# The nature of the fungal cargo induces significantly different temporal programmes of macrophage phagocytosis

**DOI:** 10.1016/j.tcsw.2022.100082

**Published:** 2022-10-13

**Authors:** María Fernanda Alonso, Judith M. Bain, Fiona M. Rudkin, Lars P. Erwig, Alistair J.P. Brown, Neil A.R. Gow

**Affiliations:** a The Aberdeen Fungal Group, School of Medicine, Medical Sciences & Nutrition, Institute of Medical Sciences, University of Aberdeen, Foresterhill, Aberdeen AB25 2ZD, UK; b Medical Research Council Centre for Medical Mycology, University of Exeter, Geoffrey Pope Building, Stocker Road, Exeter EX4 4QD, UK

**Keywords:** Cell wall, Immune recognition, Phagocytosis, Macrophage, Medical mycology

## Abstract

Phagocytosis is an essential component of our immune defence against fungal pathogens. Differences in the dynamics of phagocyte migration, recognition, uptake and phagolysosome maturation are dependent on the characteristics of the fungal cargo, and in particular to differences in cell wall composition and cellular morphology. However, studies that have focused on phagocyte interactions with individual fungal species have not enabled comparisons in the kinetics of these interactions to be made between these different species. We therefore used live cell video microscopy to examine the temporal dynamics of phagocytosis for a range of fungal cargoes by thioglycollate–elicited peritoneal macrophages from C57BL/6 mice. Uniform populations of macrophages were challenged at the same time with yeast cells of *Candida albicans*, *Candida glabrata, Saccharomyces cerevisiae* and *Cryptococcus neoformans* (wild–type and an acapsular mutant, *cap*59Δ), and spores of *Aspergillus fumigatus* and *Mucor circinelloides* to enable standardized comparative interactions to be quantified from different stages of phagocytosis*.* Differences in the rate of uptake of fungal cells varied by up to 26–fold, whilst differences in time to induce phagosome acidification varied by as much as 29–fold. Heat-killing or opsonizing the fungal targets markedly affected the kinetics of the interaction in a species–specific manner. Fungal and macrophage killing assays further revealed cargo–specific differences in phagocytosis and diversity in fungal evasion mechanisms. Therefore, simultaneous assessment of the interaction of macrophages with different fungal pathogens highlighted major differences in the kinetics and growth responses during fungus–phagocyte interactions that are likely to impact on pathogenesis and virulence.

## Highlights

• The rate of uptake of fungal cells by macrophages varies by up to 26-fold between different pathogenic fungal species.
• Phagosome acidification times for macrophages varies by as much as 29-fold for different pathogenic fungi.
• Heat-killing affects the kinetics of the interaction with macrophages in a species-dependent manner.

## 1 Introduction

Fungal diseases represent a significant and growing public health challenge (Brown et al., 2012, Fisher et al., 2020, Gow et al., 2022, Case et al., 2022). Every year approximately 3 million people acquire systemic fungal infections, of which approximately half die (Brown et al., 2012). The majority of life-threatening fungal infections are caused by species belonging to one of four genera: *Cryptococcus*, *Candida*, *Aspergillus* and *Pneumocystis* although infections caused by members of the order Mucorales are also frequently reported (Bongomin et al., 2017, Brown et al., 2012) and are often associated with various co-morbidities (Raut, 2021, Hoenigl et al., 2022).

Most pathogenic fungal species pose a particularly serious risk in immunocompromised individuals (Riley, 2021). This highlights the efficacy of our intact host defences in clearing challenges arising from regular encounters with environmental fungi and the ability of our immune system to establish a homeostatic relationship with our mycobiota (Iliev, 2022). Phagocytosis by macrophages and neutrophils is a key element of the innate host defence response to invading fungal pathogens (Lewis et al., 2012, Rudkin et al., 2013, Bain et al., 2021, Godoy et al., 2022). The process of phagocytosis can be considered to comprise five progressive stages: migration and chemotaxis, target recognition, target internalization, phagosome maturation and pathogen degradation. The dynamics of this process are critically dependent on the physical and chemical properties of the particle to be internalized, and in particular on the composition of the cell wall, which is a major source of ligands for the immune receptors integral to fungal recognition (Gow et al., 2012; Erwig and Gow, 2016, Vendele et al., 2020, Yadav et al., 2020, Briard et al., 2021).

Collectively, fungal cells represent highly diverse targets for phagocytes because they differ in shape and size and cell wall composition (Erwig and Gow, 2016, Lewis et al., 2012 Bain et al., 2021). Although the composition of the inner cell wall for most fungal cells contains some conserved polysaccharides (in particular chitin and β-1,3 glucan), there are important differences in outer cell wall composition and cell morphology. Consequently there are likely to be significant differences in induced immune defence mechanisms in relation to the various groups of clinically relevant fungal pathogens (Erwig and Gow, 2016, Gow et al., 2017, Yadav et al., 2020, Gow and Lenardon, 2022). Spherical conidia of *Aspergillus* spp. conidia and ellipsoidal cells of *Candida* spp. yeast cells are small enough to be readily engulfed whilst efficient phagocytosis is compromised by cellular enlargement of *Cryptococcus* yeast cells to form Titan cells and the ability of *Coccidioides* to form large spherules (Erwig and Gow, 2016). Filamentous fungal cells and the hyphal form of *C. albicans* can also present problems for phagocyte engulfment and phagocytes may have to fold the hypha (McKenzie et al., 2010, Bain et al., 2021) or restrict attack to engulf only a portion of the hypha tube, within a sealed tubular phagosome (Maxson et al, 2018). The outer surface of the cell wall of fungal pathogens is also highly variable. *Cryptococcus* spp. yeast cells are covered by a hydrophobic polysaccharide capsule comprising glucuronoxylomannan and galactoxylomannan, whereas *Candida* spp. yeast cells expose highly mannosylated proteins. *Aspergillus* spp. have a range of polysaccharides including galactomannan, galactosaminoglycan and α-1,3 glucan in their outer wall (Erwig and Gow, 2016). These differences in cell wall chemistry condition the interactions of different fungal pathogens with immune cells of the infected host (Leopold Wager et al., 2016, Valsecchi et al., 2017, Yadav et al., 2020).

Various research laboratories have focussed on the effects of specific fungal attributes upon different stages of the phagocytic process in order to understand which factors determine effective clearance and what mechanisms are used by different fungi to overcome the hostile environment of the phagosome (reviewed in Erwig and Gow, 2016). Most studies have addressed these processes for individual fungi and specific mutants (e.g. cell wall mutants) using a wide array of technical approaches and immunological assays. However, differences in methodology and experimental conditions (e.g. macrophage type, culture media, fungus/phagocyte stimulation ratios and incubation conditions and times) compromise direct comparisons between the interactions of immune cell types with different fungi.

In the present study we have evaluated the effects of different fungal cargoes on the temporal dynamics of phagocytosis by one type of primary macrophage. Live cell imaging of phagocytosis was performed in parallel for six fungal species: *Candida albicans*, *Candida glabrata*, *Aspergillus fumigatus*, *Cryptococcus neoformans* (including wild–type and acapsular mutant (*cap*59Δ) strains), *Saccharomyces cerevisiae* and *Mucor circinelloides*. This panel includes species of high and low virulence and wide phylogenetic diversity. The effects of fungal cell viability and opsonisation were also explored. We describe dramatic differences in the rates of engulfment and phagosome acidification for different fungal cargoes and suggest that these differences are instrumental in determining the outcomes of interactions with innate immune cells.

## 2 Materials

### 2.1 Fungal strains and growth conditions

Fungal strains used in this study are listed in Table 1. All strains were obtained from glycerol stocks stored at −80 °C. *S. cerevisiae*, *C. albicans* and *C. glabrata* were grown on YPD plates [1 % yeast extract (Oxoid), 2 % mycopeptone (Oxoid), 2 % d–glucose and 1.5 % Technical agar in distilled H_2_O]. *C. neoformans* wild–type and *cap*59Δ acapsular mutant strains were grown on YPD plates [1 % Bacto^TM^ yeast extract (BD Bioscience), 2 % Bacteriological Peptone, 2 % d-glucose and 1.5 % Technical agar in distilled H_2_O]. Plates were incubated at 30 °C until colonies formed and were then maintained at room temperature. *A. fumigatus* and *M. circinelloides* were grown on potato dextrose agar (PDA) slants (BD Bioscience) for 4–5 days in the dark at 37 °C and room temperature, respectively.

**Table 1:** Fungal strains used in this study.

Organism	Strain (name)	Genotype	Ref
*S. cerevisiae*	S288c	MATα SUC2 *gal2 mal2 mel flo1 flo8-1 hap1 ho bio1 bio6*	(Mortimer and Johnston, 1986)
*C. albicans*	SC5314	wild-type	(MacCallum et al., 2009)
*C. glabrata*	SCS71182B	wild-type	(Odds et al., 2007)
*C. neoformans*	H99	wild-type	(Perfect et al., 1980)
*C. neoformans*	TYCC33	*ade2 cap59::ADE*	(Chang and Kwon-Chung, 1994)
*A. fumigatus*	NIH 5233	wild-type	(Chaparas and Kim, 1988)
*M. circinelloides f. lusitanicus*	CBS 277.49	wild-type	(Li et al., 2011a, Li et al., 2011b)

### 2.2 Fungal preparations

Overnight colonies of *S. cerevisiae*, *C. albicans*, *C. glabrata* and *C. neoformans* wild–type and *cap*59Δ mutant were pre-cultured in 5 mL volumes of YPD and incubated for 24 h at 30 °C, 200 rpm. Fresh *A. fumigatus* and *M. circinelloides* spores were collected in PBS containing 0.1 % Tween-20 and filtered through a 40 µm cell strainer to remove hyphal fragments. Cells and spores were washed twice in PBS (6000 rpm, 3 min) and counted with a haemocytometer.

For experiments with non–viable fungi (used to prevent adaptation and growth and therefore changes occurring in the cell surface composition), 1x10^8^ fungal cells were killed at 65 °C for 30 min (*S. cerevisiae*, *C. albicans*, *C. glabrata* and *C. neoformans* wild–type and *cap*59Δ mutant) or 60 min (*A fumigatus* and *M. circinelloides*) and stored at 4 °C. Lack of viability was confirmed by plating heat-treated cells on rich agar.

### 2.3 Thioglycollate-elicited peritoneal mouse macrophages

C57BL/6 female mice were used as a source of peritoneal macrophages and were obtained from specific pathogen–free facilities at the University of Aberdeen and used at ∼ 10–14 weeks of age. Thioglycollate-elicited peritoneal macrophages were obtained from sacrificed mice 3–4 days after an intraperitoneal injection of 1 mL 3 % Brewer’s thioglycollate broth (BD Bioscience). Cells were harvested by flushing the peritoneal cavity with 10 mL ice–cold sterile 5 mM EDTA in phosphate–buffered saline (PBS) and then washed 2 times (400 g, 10 min) with RPMI 1640 Glutamax (Life Technologies) supplemented with 10 % (v/v) heat–inactivated foetal calf serum (Sigma), 200 U/mL penicillin/streptomycin (Sigma) and 10 mM HEPES (Life Technologies). For phagocytosis assays, 1.5x10^5^ cells/well were seeded onto 8–well µ–slides (ibiTreat surface, ibidi). For killing assays, 5x10^4^ cells/well were seeded onto 96-well plates filter bottom plates (MultiScreenHTS HA Filter Plate, 0.45 µm, MSHAS4510, Merck). Cells were incubated for 20–24 h at 37 °C with 5 % CO_2_, after which non–adherent cells were removed by 2 washes with supplemented RPMI 1640 medium.

### 2.4 Opsonisation of Candida cells

*C. albicans* and *C. glabrata* were opsonized with AB119 and AB140, human anti–*Candida* monoclonal antibodies developed in house (Rudkin et al., 2018). Briefly, 2.5x10^7^ stationary phase pre–washed yeast cells were incubated with 50 µg/mL AB119 or AB140 for 45 min at room temperature in PBS before co–incubation with macrophages.

For immunofluorescence, cells were fixed in 4 % paraformaldehyde for 30 min at room temperature, washed with PBS and blocked with 1.5 % goat serum for 30 min. Cells were incubated with primary antibody [AB119 or AB140], washed and incubated with secondary antibody [goat anti–human IgG Alexa Fluor 488 (Molecular Probes)]. Antibody incubations were performed for 1 h at room temperature.

### 2.5 Live cell imaging phagocytosis assays

Prior to experiments, macrophage-containing wells were replenished with supplemented RPMI 1640 medium containing 50 nM LysoTracker Red (LTR) DND-99 (Invitrogen) and incubated for an hour at 37 °C, 5 % CO_2_. Standard phagocytosis assays were performed as described previously (Lewis et al., 2013). Briefly, fungal cells (live, heat–killed or opsonized) were combined with adhered macrophages at a multiplicity of infection (MOI) of 1:1 immediately prior to image acquisition. Video microscopy phagocytosis assays were performed using an UltraVIEW VoX spinning–disk microscope (PerkinElmer) with an environmental control chamber. Single-plane images were captured using a Nikon Plan Fluor 40X oil objective (NA 1.30) or a Nikon X60 Plan Apochromat VC oil objective (NA 1.40) every 2 min for up to 6 h. Every effort was taken to minimise any between experiment conditions and to ensure that conditions for the preparations of cargo cells and macrophages were reproduced as precisely as possible.

### 2.6 Analysis of live cell video microscopy movies

Image analysis was performed using Volocity 6.3 software (Improvision, PerkinElmer). Measurements taken include macrophage migration, rates of fungal uptake (% engulfed fungi over time), % phagocytic macrophages (% macrophages that engulfed at least 1 fungal cell), average number of fungal cells taken up per macrophage and time to phagosomal acidification assessed by LTR localization to individual phagosomes. Phagosomes were scored as LTR positive upon the appearance of a halo of fluorescence, which was verified in representative examples as being above background by measuring a profile of intensity with Volocity line tool. Time to acidification was calculated by subtracting the time point at which a fungal cell was fully enclosed from the time point at which a LTR positive halo was observed. Representative movies of fungus-macrophage interactions as described in the text are provided in the Supplementary materials (S-Video 1–6).

### 2.7 Macrophage damage assay

The release of lactate dehydrogenase (LDH) into the culture supernatant was monitored as a measure of host cell damage. Thioglycollate-elicited peritoneal macrophages seeded in 96–well plates filter bottom plates (MultiScreenHTS HA Filter Plate, 0.45 µm, MSHAS4510, Merck) were infected with live fungal cells at an MOI of 1:1 and incubated at 37 °C, 5 % CO_2_ for 16 h. As a maximum damage control, macrophages were treated with 2 % Triton X-100 solution. As a baseline lysis control macrophages were incubated with medium only. After co––incubation culture supernatants were collected and the amount of LDH was determined using a Cytotoxicity Detection Kit (Roche Applied Science) according to the manufacturer’s instructions. LDH activity was determined spectrophotometrically by measuring the optical density (OD) of wells at 492 nm and correlated to macrophage damage by the following equation: % macrophage damage= (OD_492_ sample – average OD_492_ baseline lysis control)/ (OD_492_ maximum damage control – average OD_492_ baseline lysis control) x100.

### 2.8 Macrophage fungicidal assay

Thioglycollate-elicited peritoneal macrophages seeded in 96–well filter bottom plates (MultiScreenHTS HA Filter Plate, 0.45 µm, MSHAS4510, Merck) were infected with fungal cells at an MOI of 1:1 and incubated at 37 °C, 5 % CO_2_ for 16 h. These conditions were used as standard in all challenge experiments. As a macrophage lysis control, macrophages were incubated with medium only. As a control of maximum metabolic activity, fungal cells were incubated with medium only. After co––incubation culture supernatants were discarded and macrophages were lysed with sterile ice–cold milliQ H_2_O/0,025 % Triton X-100. Lysis buffer was discarded and the remaining fungal cells were incubated with 200 µl/well XTT-menadione solution [200 µg/ml XTT (Sigma)/4,3 µg/ml menadione (Sigma)]. Plates were incubated at 37 °C, 5 % CO_2_ for 2–4 h depending on color development of positive controls. Upon color development, optical density (OD) of wells was measured at 450 nm and correlated to fungal damage by the following equation: % fungal damage= (OD_450_ sample – average OD_492_ baseline lysis control)/ (OD_492_ fungi only control – average OD_492_ baseline lysis control) x100.

### 2.9 Statistical analyses

Datasets were tested using the D’Agostino & Pearson omnibus normality test to determine if they were well modelled by a Gaussian distribution. For normally distributed data, mean values and standard deviations were calculated. Statistical significance was assessed by one-way analysis of variance (ANOVA) with Bonferroni’s Multiple Comparison Tests or unpaired two–tailed Student’s t–tests, as appropriate. For non– normally distributed data, median values and interquartile ranges were calculated. Statistical significance was assessed by Kruskal-Wallis test followed by Dunn’s multiple comparison test. In all cases a P value of less than 0.05 was considered significant.

### 2.10 Ethics statement

All animal procedures were conducted in accordance with the terms and conditions of the United Kingdom Home Office licence 70/8073 for research on animals and the University of Aberdeen ethical committee.

## 3 Results

### 3.1 Enhanced macrophage kinesis in response to *C. Albicans* and *C. Glabrata*

**Fig. 1:**
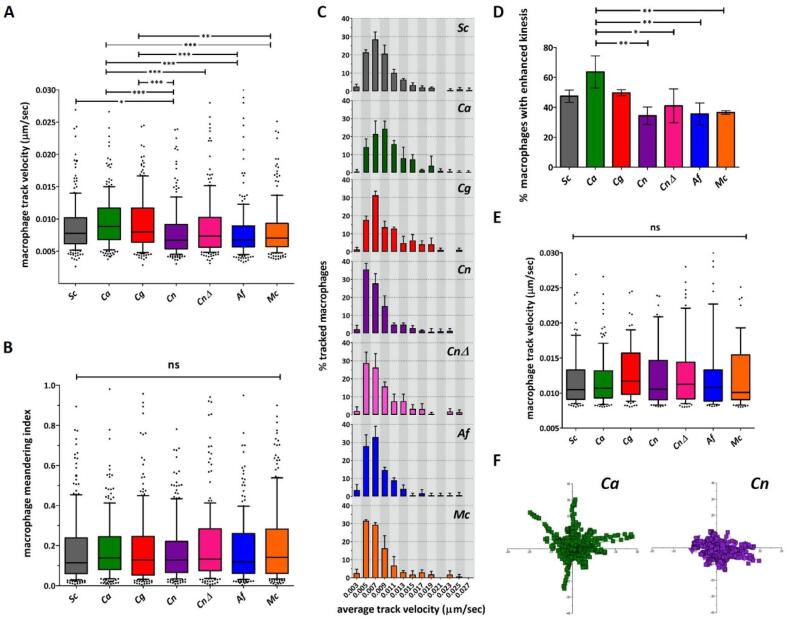
A proportion of macrophages exhibit early enhanced kinesis in response to *C. albicans* and *C. glabrata*. [A-B] Macrophages co-incubated with different fungal species were tracked for the first 60 min of image acquisition. Mean track velocities and meandering indexes (track’s deviation from a straight line) were calculated. Macrophages showed higher average macrophage mean track velocities [A] when co-incubated with *C. albicans* and *C. glabrata*, but the responses were not more directional (low meandering indexes, no differences between fungal targets) [B]. Data are represented as box and whiskers [IQR (boxes), 10–90 percentile (whiskers), median (horizontal line) and outliers (dots)] of 200 individual macrophages from 3 biologically independent replicates. Statistical significance was assessed by Kruskal-Wallis test followed by Dunn’s multiple comparison test. [C-D] Larger proportion of macrophages exhibited higher track velocities when co-incubated with *C. albicans* and *C. glabrata*. Frequency distribution histograms of macrophage track velocities show shift towards higher velocities [C] and higher percentage of macrophages show enhanced kinesis (higher track velocities than non-infected macrophage control) [D] when co-incubated with *C. albicans* and *C. glabrata*. Data are represented as mean ± SD of 3 independent biological replicates. Histogram bin size = 0.002 µm/*sec*. Statistical significance was assessed by one-way ANOVA test followed by Bonferroni’s multiple comparison test. [E] Macrophages showing enhanced kinesis did not exhibit differences in average mean track velocity. Data are represented as box and whiskers [IQR (boxes), 10–90 percentile (whiskers), median (horizontal line) and outliers (dots)] of selected macrophages from 3 biologically independent replicates. Statistical significance was assessed by Kruskal–Wallis test followed by Dunn’s multiple comparison test. [F] Representative tracking diagrams of macrophages co-incubated with [i] *C. albicans* and [ii] *C. neoformans* wild type strain. Each track represents the movement of an individual macrophage relative to its starting position. Symbols indicate the location of macrophages at 2 min intervals for 1 h. *Sc*: *S. cerevisiae*, *Ca*: *C. albicans*, *Cg*: *C. glabrata*, *Cn*: *C. neoformans* (wild-type), *Cn*Δ: *C. neoformans cap*59Δ (acapsular mutant), *Af*: *A. fumigatus*, *Mc*: *M. circinelloides.* *P ≤ 0.05, **P ≤ 0.01, ***P ≤ 0.001, ns = P > 0.05.

Phagocyte migration towards fungal cells is the initial step in the phagocytic process. Analysis of the initial 60 min of macrophage movement towards different fungal targets, revealed that macrophages exhibited higher average mean track velocities when co–incubated with *C. albicans* [median (IQR) = 0.0088 µm/*sec* (0.0069–0.012 µm/*sec*)] and *C. glabrata* [median (IQR) = 0.0080 µm/*sec* (0.0064–0.012 µm/*sec*)] compared to *C. neoformans* wild type [median (IQR) = 0.0067 µm/*sec* (0.0054–0.0092 µm/*sec*)] and *cap*59Δ strains [median (IQR) = 0.0073 µm/*sec* (0.0056–0.010 µm/*sec*)], *A. fumigatus* [median (IQR) = 0.0067 µm/*sec* (0.0057–0.0089 µm/*sec*)] and *M. circinelloides* [median (IQR) = 0.0070 µm/*sec* (0.0057–0.0093 µm/*sec*)]. Average macrophage mean track velocity when co-incubated with *S. cerevisiae* showed an intermediate value [median (IQR) = 0.0078 µm/*sec* (0.0061–0.010 µm/*sec*)] (Fig. 1 A).

To evaluate how directional the movement of macrophages was towards different targets, “meandering indexes” (ME) were calculated. This index provided a measure of a track’s deviation from a straight line. An ME value of 1 indicated the track was a perfect straight line, lower values indicated higher degrees of cell meandering. No significant differences in meandering indexes were observed for macrophages co–incubated with different fungal pathogens (Fig. 1B).

Frequency distribution histograms of macrophage track velocities showed shift towards higher velocities when co-incubated with *C. albicans* and *C. glabrata* (Fig. 1 C). The percentage of macrophages exhibiting higher mean track velocities than a non–infected macrophage control was calculated. A higher percentage showed enhanced kinesis when co–incubated with *C. albicans* (mean ± SD = 63 % ± 11 %) compared to *C. neoformans* wild type (mean ± SD = 34 % ± 6 %) and *cap*59Δ (mean ± SD = 41 % ± 11 %) strains, *A. fumigatus* (mean ± SD = 36 % ± 7 %) and *M. circinelloides* (mean ± SD = 37 % ± 1 %). Though not statistically significant at P = 0.05, the same trend was observed for enhanced macrophage migration towards *C. glabrata* (mean ± SD = 50 % ± 2 %) (Fig. 1D). A comparison of average mean track velocities amongst macrophages exhibiting enhanced kinesis, revealed no differences between fungal targets (Fig. 1E). Collectively these results suggest that a larger proportion of macrophages exhibited enhanced kinesis towards *C. albicans* and *C. glabrata* than for the other fungal species examined, but that this movement was not more directional in nature.

**Fig. 2:**
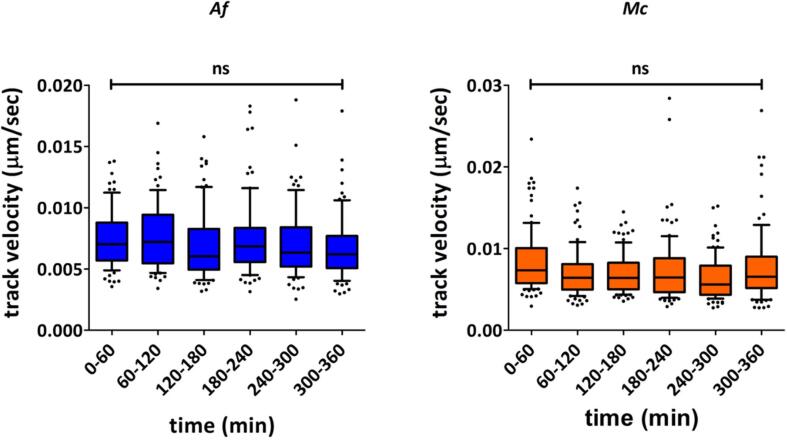
Macrophage migration was not increased in response to spore swelling and germination for *A. fumigatus* and *M. circinelloides.* Macrophages co-incubated with *A. fumigatus* and *M. circinelloides* were tracked for 360 min of image acquisition and mean track velocities were calculated. No significant differences were observed in average mean track velocities over time for the two species studied. Data are represented as box and whiskers [IQR (boxes), 10–90 percentile (whiskers), median (horizontal line) and outliers (dots)] of at least 70 individual macrophages from 3 biologically independent replicates. All times shown are in reference to start of imaging (approximately 30 min post fungal–macrophage co–incubation). Statistical significance was assessed by Kruskal-Wallis test followed by Dunn’s multiple comparison test. *Af*: *A. fumigatus*, *Mc*: *M. circinelloides*; ns = P > 0.05.

Since *A. fumigatus* and *M. circinelloides* spores exhibit a lag phase before initiating a multi–step process of germination (isotropic growth, followed by a phase of polarised growth and hyphal extension), the analysis of macrophage migration was extended to 360 min. This experimental design anticipated the possibility of enhanced macrophage migration towards swollen spores/germ tubes. However, no significant differences in average macrophage mean track velocities were observed over the 360 min of analysis (Fig. 2).

### 3.2 Fungal cells exhibit distinct engulfment, phagosome maturation and killing rates

**Fig. 3:**
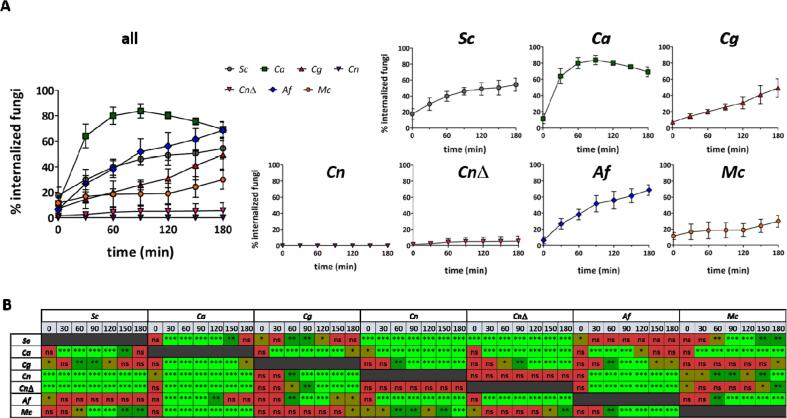
Primary macrophages exhibit markedly different engulfment rates towards different fungal targets. Engulfment rates of different fungal targets by thioglycollate–elicited peritoneal macrophages were determined following live cell imaging. [A] Percentages of fungal cells inside macrophages relative to total number of fungal cells per field of view were calculated every 30 min for 180 min. Data are represented as mean ± SD from 4 biologically independent replicates. For each independent replicate at least two fields of view were analysed so that at least 60 fungal cells were quantified. All times shown are in reference to start of imaging (approximately 30 min post fungal–macrophage co–incubation). [B] Statistical significance was assessed by one–way ANOVA test followed by Bonferroni’s multiple comparison test. *Sc*: *S. cerevisiae*, *Ca*: *C. albicans*, *Cg*: *C. glabrata*, *Cn*: *C. neoformans* (wild-type), *Cn* wild-type: *C. neoformans cap*59Δ (acapsular mutant), *Af*: *A. fumigatus*, *Mc*: *M. circinelloides.* *P ≤ 0.05 (brown), **P ≤ 0.01 (dark green), ***P ≤ 0.001 (light green), ns = P > 0.05 (red). (For interpretation of the references to color in this figure legend, the reader is referred to the web version of this article.)

Engulfment rates represent the combined result of the dynamics of macrophage migration towards and recognition of its phagocytic target. At the onset of the experiment macrophages had taken up a larger proportion of *S. cerevisiae* cells than of any other fungus (Fig. 3). The initial proportion of internalized *C. glabrata* (mean ± SD = 7 % ± 1 %), *C. neoformans* wild type (mean ± SD = 0 % ± 0 %) and *cap*59Δ strains (mean ± SD = 2 % ± 1 %) and *A. fumigatus* (mean ± SD = 7 % ± 4 %) was at least half that for *S. cerevisiae* cells (mean ± SD = 18 % ± 7 %).

This rank order changed in the first 30 min post initiation of imaging. By this time, uptake of *C. albicans* cells (mean ± SD = 64 % ± 9 %) was at least double that for any other fungus [*S. cerevisiae* (mean ± SD = 30 % ± 8 %); *C. glabrata* (mean ± SD = 15 % ± 3 %); *C. neoformans* wild type (mean ± SD = 0 % ± 0 %) and *cap*59Δ (mean ± SD = 3 % ± 2 %) strains; *A. fumigatus* (mean ± SD = 27 % ± 6 %) and *M. circinelloides* (mean ± SD = 17 % ± 10 %)] (Fig. 3). This increase in uptake coincided with the average time for the initiation of evagination of germ tubes by *C. albicans*. By 60 min after initialisation of video analysis, the majority of *C. albicans* cells had been engulfed (mean ± SD = 80 % ± 7 %). By 90 min after initialisation of video analysis, internalized and non–internalized *C. albicans* hyphal cells exceeded 20 µm in length. Longer hyphae were less frequently engulfed and hypha formation allowed some cells to escape the phagocyte, thus accounting for the decrease in percentage of internalized fungal cells observed after this time point (Fig. 3). These data were in accord with, and extend, previous observations (McKenzie et al., 2010, Lewis et al., 2012, Bain et al., 2021).

**Fig. 4:**
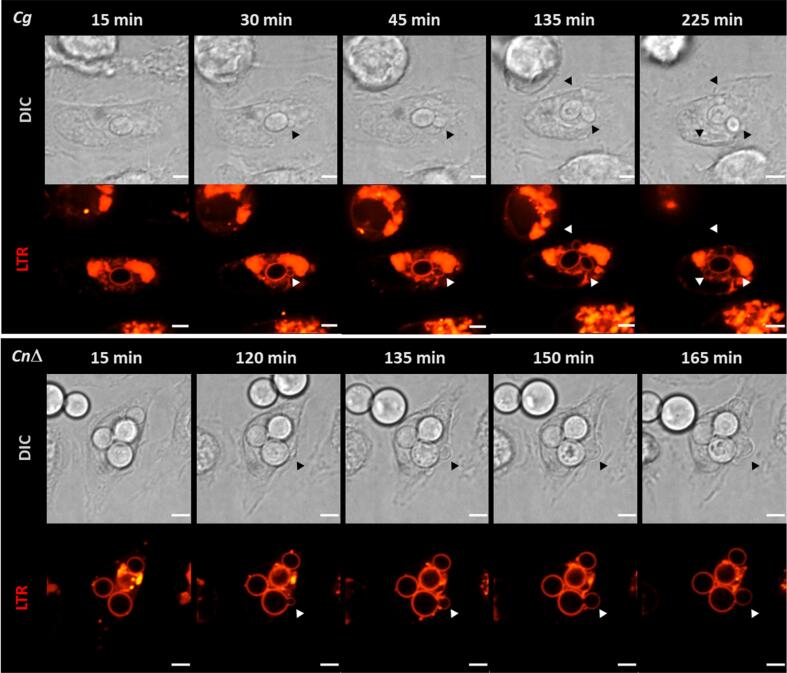
Intraphagosomal proliferation of *C. glabrata* and *C. neoformans cap*59Δ. Selected time point frames from live cell imaging movies showing intraphagosomal proliferation of *C. glabrata* and *C. neoformans cap*59Δcells. Macrophage acidic compartments were stained with LTR (red). Selected times are indicated in upper panel. All times shown are in reference to start of imaging (approximately 30 min post fungal–macrophage co–incubation). Arrowheads indicate new cells budding from previously engulfed fungal cells within the phagosomal compartment. Scale = 4 µm. (For interpretation of the references to color in this figure legend, the reader is referred to the web version of this article.)

*C. glabrata* uptake increased steadily over the time analysed. At the start of imaging the percentage of engulfed *C. glabrata* cells was less than half that of *S. cerevisiae*; but by 150 min the percentages were comparable [*C. glabrata* (mean ± SD = 41 % ± 11 %); *S. cerevisiae* (mean ± SD = 51 % ± 9 %)] (Fig. 3). Most of the uptake events of *C. glabrata* occurred at later time points (after 120 min) and correlated with increased fungal doubling. *C. glabrata* proliferation occurred outside and inside phagocytes (Fig. 4).

The *C. neoformans* wild–type strain was not taken up during the time span analysed in the experiment (Fig. 3) but the acapsular mutant was infrequently internalized. Few uptake events occurred at early time points (Fig. 3). As described for *C. glabrata*, intracellular proliferation was observed for internalized *C. neoformans cap*59Δ (Fig. 4).

*A. fumigatus* phagocytic uptake increased consistently over time. During the first 60 min of image acquisition uptake of *A. fumigatus* was half that of *C. albicans*. However, by 180 min the percentage of engulfed *A. fumigatus* cells (mean ± SD = 69 % ± 6 %) was comparable to the percentage of engulfed *C. albicans* cells (mean ± SD = 69 % ± 6 %) (Fig. 3).

In contrast, few *M. circinelloides* spores were internalized during the first 150 min of image acquisition (mean ± SD = 24 % ± 8 %). By this time, the percentage of engulfed *M. circinelloides* cells was half for engulfed *C. albicans* cells (mean ± SD = 76 % ± 3 %) (Fig. 3).

**Fig. 5:**
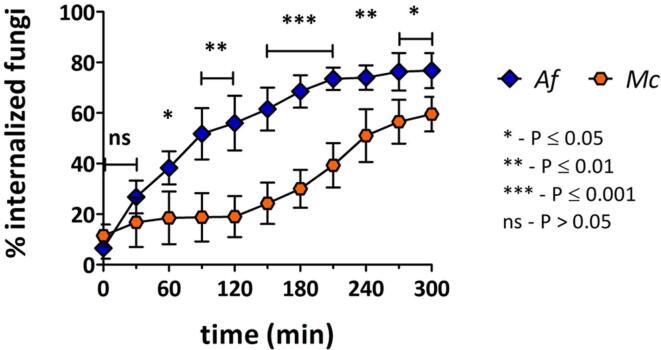
Primary macrophages exhibit markedly different engulfment dynamics towards *A. fumigatus* and *M. circinelloides* spores. An extended analysis of macrophage engulfment of *A. fumigatus* and *M. circinelloides* spores was performed following live cell imaging. Percentages of fungal cells inside macrophages relative to total number of fungal cells per field of view were calculated every 30 min for 300 min. While *A. fumigatus* uptake rates increased steadily over time, engulfment of *M. circinelloides* exhibited a prolonged lag phase. For *M. circinelloides* higher uptake rates were observed after 150–180 min of image acquisition. Data are represented as mean ± SD from 4 biologically independent replicates. For each independent replicate at least two fields of view were analysed so that at least 60 fungal cells were quantified. All times shown are in reference to start of imaging (approximately 30 min post fungal–macrophage co–incubation). Statistical significance was assessed by unpaired two–tailed Student’s *t*-test. *Af*: *A. fumigatus*, *Mc*: *M. circinelloides*;*P ≤ 0.05, **P ≤ 0.01, ***P ≤ 0.001, ns = P > 0.05.

Since *A. fumigatus* and *M. circinelloides* undergo a slower, multi–step process of germination, the analysis of macrophage engulfment was extended to 300 min (Fig. 5). Extended analysis of *A. fumigatus* uptake dynamics showed that internalization ceased approximately 240 min post start of imaging, which coincided with the initiation of germ tube emergence. In contrast, uptake of *M. circinelloides* increased dramatically after 180 min. By 300 min over half of *M. circinelloides* cells had been internalized (mean ± SD = 60 % ± 7 %). Increased uptake of *M. circinelloides* cells at later time points correlates with spore swelling.

**Fig. 6:**
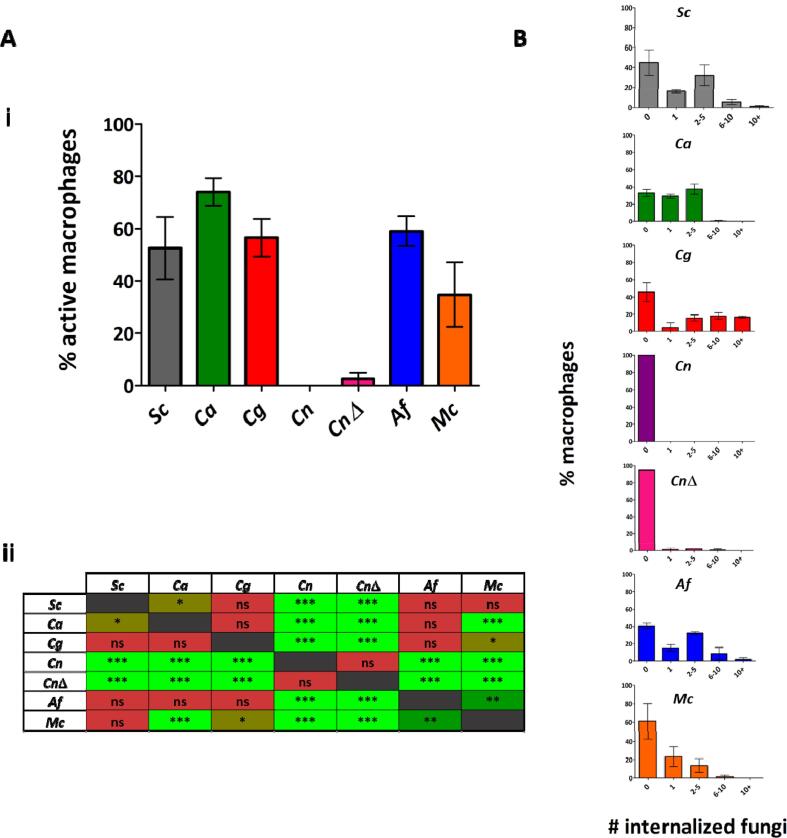
Higher percentages of phagocytic macrophages are observed in co–incubation with *C. albicans*, *C. glabrata* and *A. fumigatus*. [A] i- Percentages of macrophages that had internalized at least one fungal cell after 180 min of image acquisition were determined following live cell microscopy. A majority of macrophages were actively phagocytic towards *C. albicans*, *C. glabrata*, *A. fumigatus* and *S. cerevisiae*. Data are represented as mean ± SD from 4 biologically independent replicates. For each independent replicate at least five fields of view were analysed so that at least 40 macrophages were quantified. ii- Statistical significance was assessed by one–way ANOVA test followed by Bonferroni’s multiple comparison test. [B] Number of internalized fungal cells per macrophage were determined after 180 min of image acquisition. A significantly higher percentage of macrophages engulfed over 10*C. glabrata* cells than of other fungal species. Data are represented as mean ± SD from 4 biologically independent replicates. For each independent replicate at least five fields of view were analysed so that at least 40 macrophages were quantified. *Sc*: *S. cerevisiae*, *Ca*: *C. albicans*, *Cg*: *C. glabrata*, *Cn*: *C. neoformans* (wild-type), *Cn* wild-type: *C. neoformans cap*59Δ (acapsular mutant), *Af*: *A. fumigatus*, *Mc*: *M. circinelloides.* *P ≤ 0.05 (brown), **P ≤ 0.01 (dark green), ***P ≤ 0.001 (light green), ns = P > 0.05 (red). (For interpretation of the references to color in this figure legend, the reader is referred to the web version of this article.)

Analysis of the percentage of actively phagocytic macrophages (i.e. macrophages that internalized at least one fungal cell) when co–incubated with the different fungal targets, revealed that a higher proportion of macrophages were active towards *C. albicans*, *C. glabrata* and *A. fumigatus* than towards *C. neoformans* wild type and *cap*59Δ strains and *M. circinelloides* (Fig. 6 A).

Analysis of the number of fungal cells internalized by macrophages (Fig. 6 B) showed a significantly higher percentage of macrophages contained over 10*C. glabrata* cells (mean ± SD = 17 % ± 1 %), with some macrophages containing over 20*C. glabrata* cells. The percentage of macrophages that internalized over 10 fungal cells for the other species was negligible.

**Fig. 7:**
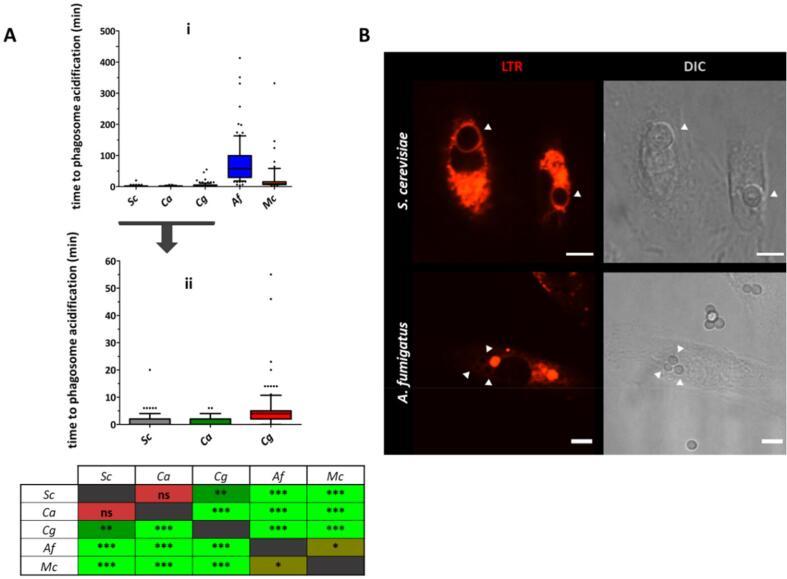
Acidification of *A. fumigatus* and *M. circinelloides* containing phagosomes was markedly delayed in comparison to other fungal targets. [A] Time taken for each fungal–containing phagosome to acidify was defined as the time between full enclosure of the fungal cell to appearance of surrounding LTR halo. *C. albicans–*, *C. glabrata*, and *S. cerevisiae*– containing phagosomes acidified more quickly than those containing *A. fumigatus* or *M. circinelloides*. Data are represented as box and whiskers [IQR (boxes), 10–90 percentile (whiskers), median (horizontal line) and outliers (dots)] of at least 100 individual fungal cells from 3 biologically independent replicates. Statistical significance was assessed by Kruskal-Wallis test followed by Dunn’s multiple comparison test. [B] Representative images extracted from live cell movies showing differences in LTR signal for *S. cerevisiae*–containing phagosomes and *A. fumigatus*–containing phagosomes. Arrows indicate LTR positive phagosomal compartments. Scale = 6 µm. *Sc*: *S. cerevisiae*, *Ca*: *C. albicans*, *Cg*: *C. glabrata*, *Af*: *A. fumigatus*, *Mc*: *M. circinelloides.* *P ≤ 0.05 (brown), **P ≤ 0.01 (dark green), ***P ≤ 0.001 (light green), ns = P > 0.05 (red). (For interpretation of the references to color in this figure legend, the reader is referred to the web version of this article.)

The next step in the phagocytic process is the maturation of the newly formed phagosome. Early phagosomes have similar composition to the extracellular milieu and lack the ability to kill and degrade ingested microbes. Acidification of the phagosomal compartment is an indicator of maturation and a requisite for full phagosomal microbicidal capacity. Acidification occurred promptly after engulfment for *S. cerevisiae*– [median (IQR) = 2 min (0–2 min)], *C. albicans*- [median (IQR) = 2 min (0–2 min)] and *C. glabrata-* [median (IQR) = 4 min (2–5 min)] containing phagosomes (Fig. 7A). *M. circinelloides-* and *A. fumigatus*-containing phagosomes acidified markedly more slowly [median (IQR) equals 9 min (6.5–15.5 min) and 58 min (29.5–99.5 min), respectively] (Fig. 7.A). Though intensity of LTR signal was not quantified, it was noted that positive LTR *A. fumigatus*– and *M. circinelloides*–containing phagosomes showed weaker LTR signals than *S. cerevisiae*– and *C. albicans*–containing phagosomes (Fig. 7B). Although the number of internalized *C. neoformans cap*59Δ cells was too low to include in the overall comparison, preliminary analysis of time to phagosomal acidification showed similar results to those exhibited by *S. cerevisiae*, *C. albicans* and *C. glabrata* (data not shown).

**Fig. 8:**
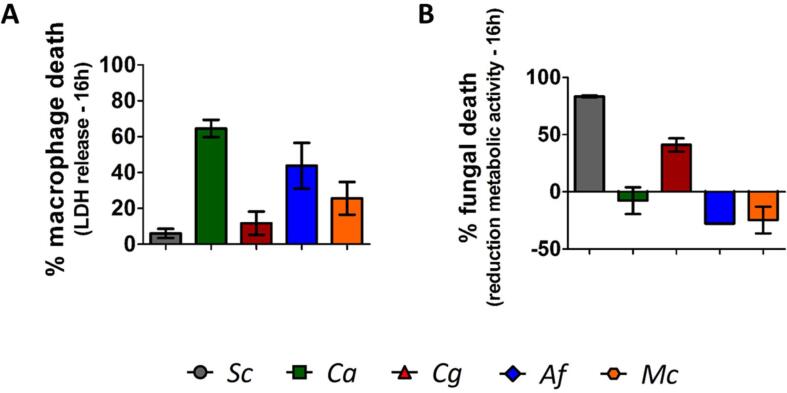
Non–filamentous fungi exhibited susceptibility to macrophage killing, while filamentous fungi showed marked capacity to damage host cells. [A] Measurements of fungal capacity to damage host cells, as assessed by LDH release from macrophages into the culture medium at 16 h post–incubation. Data are represented as mean ± SD from two biologically independent repeats. [B] Fungal metabolic activity assay showing reduction in fungal metabolic activity for *S. cerevisiae* and *C. glabrata* when co–incubated with macrophages. Data are represented as mean ± SD from two biologically independent repeats. *Sc*: *S. cerevisiae*, *Ca*: *C. albicans*, *Cg*: *C. glabrata*, *Af*: *A. fumigatus*, *Mc*: *M. circinelloides.*

The final outcome of the phagocytic process is the killing of the internalized microbe. However, several pathogens possess mechanisms to escape or delay this process. Preliminary analyses of the ability of phagocytes to kill a selected panel of these fungi and the ability of the different fungi to damage the phagocyte were performed. Fungal viability was estimated using a metabolic activity assay (XTT assay) and macrophage damage was evaluated by measuring phagocyte lactate dehydrogenase release from lysed cells. The preliminary analyses suggested that after 16 h co–incubation, primary macrophages killed internalized non–filamentous yeasts (*S. cerevisiae* and *C. glabrata*) but not filamentous fungi (*C. albicans*, *A. fumigatus* and *M. circinelloides*) (Fig. 8). Filamentous fungi, in particular *C. albicans*, showed a marked ability to damage host cells, which was not observed in non–filamentous fungi (Fig. 8).

### 3.3 Heat–killing of cells differentially affects macrophage phagocytic dynamics

Exposing fungal cells to high temperatures, results in death and concomitant exposure of inner cell wall components. Mild heat treatment therefore fixes the cell target and prevents morphological and cell wall architectural changes associated with dynamic environmental adaptations, but also results in exposure of immune ligands that would be covered in native, viable cells. The effect of these modifications on macrophage uptake dynamics was assessed.

**Fig. 9:**
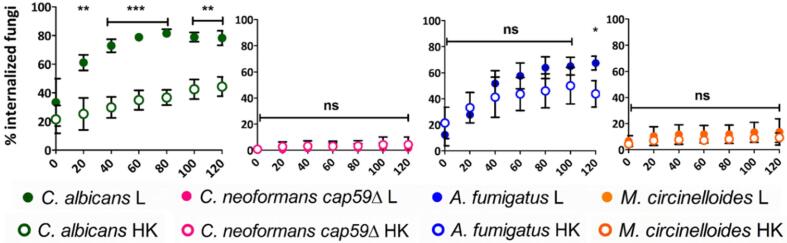
Loss of viability and alterations to fungal cell-wall architecture affect macrophage phagocytic dynamics differently for different fungal targets. Percentages of fungal cells inside macrophages relative to total number of fungal cells were calculated for selected live and heat–killed fungal targets every 20 min for 120 min. Uptake of live cells was faster for *C. albicans* and, to a lesser extent, *A. fumigatus* compared to HK cells. No differences in uptake were observed between live and HK cells for *C. neoformans cap*59Δ and *M. circinelloides*. Data are represented as mean ± SD from 3 biologically independent replicates. For each independent replicate at least four fields of view were analysed so that at least 50 fungal cells were quantified. All times shown are in reference to start of imaging (approximately 30 min post fungal–macrophage co–incubation). Statistical significance was assessed by unpaired two–tailed Student’s *t*-test. L = live cells; HK = heat–killed cells; *P ≤ 0.05, **P ≤ 0.01, ***P ≤ 0.001, ns = P > 0.05.

Engulfment of live *C. albicans* cells was significantly faster than engulfment of heat–killed (HK) *C. albicans* cells. Over twice as many live *C. albicans* cells (mean ± SD = 61 % ± 5 %) were internalized by 20 min of image acquisition, compared to HK *C. albicans* cells (mean ± SD = 25 % ± 11 %) (p ≤ 0.01). A significant difference was still observed by 120 min of image acquisition: 1.7 times more live *C. albicans* cells (mean ± SD = 78 % ± 5 %) had been engulfed compared to HK *C. albicans* cells (mean ± SD = 44 % ± 7 %) (p ≤ 0.01) (Fig. 9). This suggests that inner fungal wall components (exposed by HK treatments) did not contribute greatly to the dynamics of initial interactions with macrophages. Similarly, a higher percentage of live *A. fumigatus* cells (mean ± SD = 67 % ± 5 %) were internalized by 120 min of image acquisition, compared to the percentage of internalized HK *A. fumigatus* cells (mean ± SD = 44 % ± 10 %) (p ≤ 0.05). This difference was not observed at earlier time points (Fig. 9). These results suggest that morphological transitions, such as *C. albicans* filamentation and *A. fumigatus* swelling, are likely to be important for macrophage recognition. No differences in uptake were observed between live and HK cells for *C. neoformans cap*59Δ or *M. circinelloides* over 120 min of image acquisition (Fig. 9).

### 3.4 Opsonisation markedly affects macrophage uptake dynamics

**Fig. 10:**
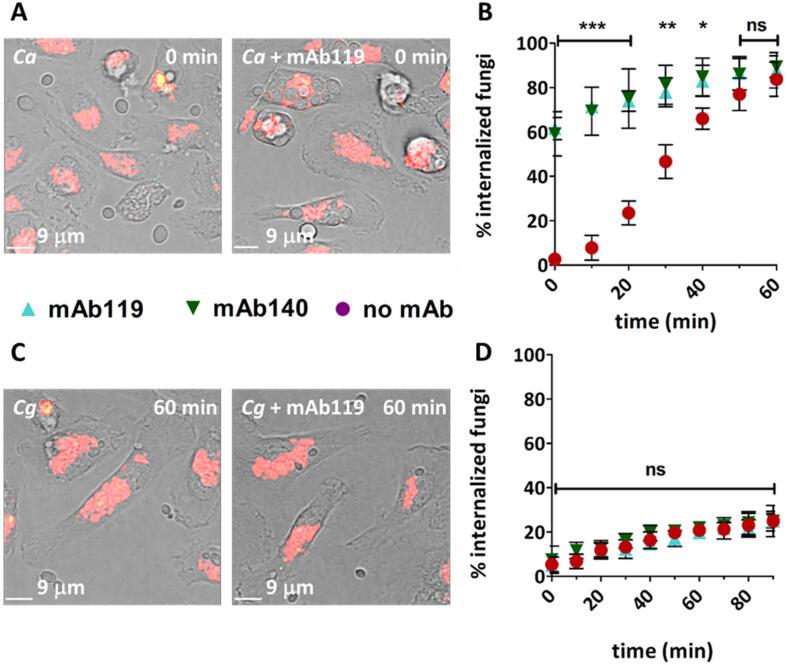
Opsonisation of *Candida* cells with pan–*Candida* monoclonal antibodies increased fungal uptake rates for *C. albicans*, but not *C. glabrata*. A and C) Representative snapshots from live cell imaging videos of primary macrophages co–incubated with *C. albicans* or *C. glabrata* cells pre–treated and not pre–treated with mAb119. Macrophages were stained with LTR to visualize acidic compartments. B and D) Percentage of fungal cells inside macrophages relative to total number of fungal cells per field of view where calculated every 10 min for 60 or 90 min. Data are represented as mean ± SD from 3 biologically independent replicates. For each independent replicate at least four fields of view were analysed so that at least 50 fungal cells were quantified. All times shown are in reference to start of imaging (approximately 30 min post fungal–macrophage co–incubation). Statistical significance was assessed by unpaired two–tailed Student’s *t*-test. *Ca*: *C. albicans*, *Cg*: *C. glabrata*, *P ≤ 0.05, **P ≤ 0.01, ***P ≤ 0.001, ns = P > 0.05.

*C. albicans* and *C. glabrata* cells were opsonized with the pan–*Candida* species monoclonal antibodies AB119 and AB140 which had been raised against *C. albicans* cell wall. (Rudkin et al, 2018). Opsonisation resulted in faster uptake of *C. albicans* yeast cells. The percentage of internalized *C. albicans* cells at start of imaging was approximately 20 times higher when opsonized with AB119 (mean ± SD = 62 % ± 5 %) or AB140 (mean ± SD = 59 % ± 10 %), compared to non–opsonized cells (mean ± SD = 3 % ± 2 %) (p ≤ 0.001). By 60 min of image acquisition, no differences in engulfment rates were observed between *C. albicans* cells opsonized with AB119 (mean ± SD = 88 % ± 8 %) or AB140 (mean ± SD = 89 % ± 5 %) and non–opsonized cells (mean ± SD = 83 % ± 8 %) (Fig. 10 A and B).

**Fig. 11:**
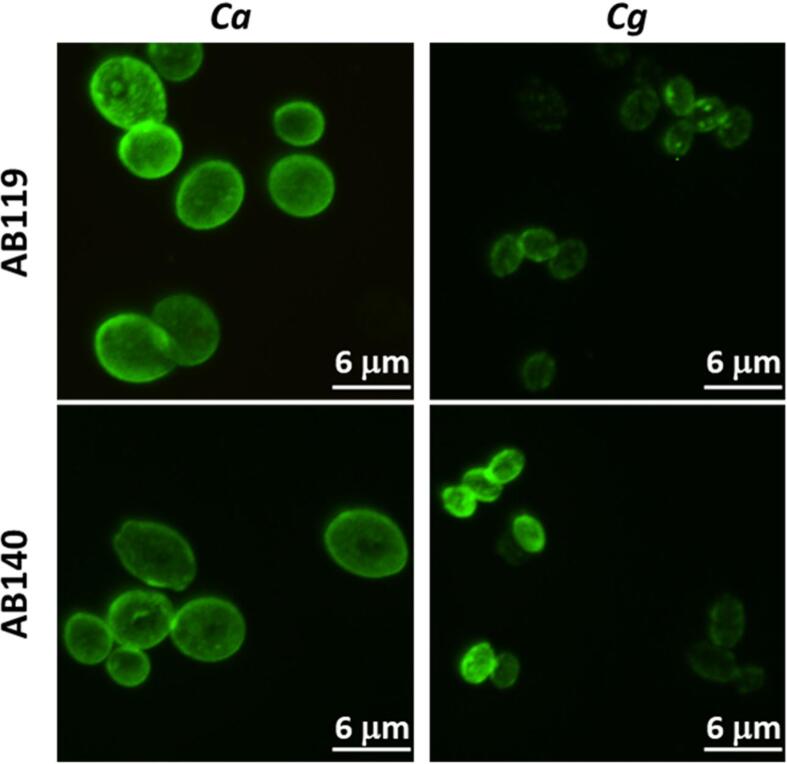
Differences in binding patterns of AB119 and AB140 to *C. albicans* and *C. glabrata*. Immunofluorescence microscopy revealed differences in binding patterns of AB119 and AB140 to *C. albicans* and *C. glabrata*. In both cases the antibody bound to the whole cell, but the stain was more heterogeneous and punctuated for *C. glabrata*. *Ca*: *C. albicans*, *Cg*: *C. glabrata.*

Contrary to the observations made with opsonised *C. albicans*, uptake rates of *C. glabrata* cells were not altered by opsonisation with AB119 or AB140 (Fig. 10 C and D) suggesting that these antibodies bound differently to these two species. Confirming this, immunofluorescence microscopy revealed differences in binding patterns of AB119 and AB140 to *C. albicans* and *C. glabrata*. In both cases the antibody bound to the whole cell, but the stain was more heterogeneous and punctuated for *C. glabrata* (Fig. 11).

## 4 Discussion

Host immune defence against fungal infections depends on the sentinel activity of phagocytic cells, such as macrophages, which recognize, internalize and degrade invading fungal cells leading to antigen presentation and subsequent activation of other parts of the immune response. The different stages of the phagocytic process (migration, engulfment, maturation and killing) were studied simultaneously for six phylogenetically diverse and medically relevant fungal pathogens using a fixed population of macrophages. Every care was taken to minimise potential variables in creating near identical conditions for the preparation of the host cargo cells and macrophages to enable within and between experimental comparisons to be made. Profound differences were observed in the dynamics of the responses towards the different fungal targets. In particular, differences in uptake of fungi varied by up to 26-fold (*C. albicans* vs *C. neoformans cap*59Δ at 30 min post initiation of imaging). Differences in average speed of phagosome acidification varied by as much as 29-fold (*S. cerevisiae* and *C. albicans* vs *A. fumigatus*). Given that no external source of complement components was added to the experimental set-up used in this work, the major differences in the kinetics of phagocytosis may be attributed to differences in wall composition and the three dimensional architecture of the fungal cell target (Erwig and Gow, 2016, Gow et al., 2017, Gow and Lenardon, 2022). The addition of exogenous complement components to the experiments would have potentially masked such differences by sterically blocking access to some externally exposed cell wall components and allowing internalization by generic receptors, like CR3 and CR4. Differential secretion of cytokines, chemokines and complement components by peritoneal macrophages in response to interaction with the different fungal targets was beyond the scope of this work and warrants further study.

The *C. albicans* outer cell wall is mainly composed of mannans, which can be recognized by multiple phagocytic immune receptors (Netea et al., 2008). The cell wall of *C. neoformans* is surrounded by a thick hydrophobic polysaccharide capsule of glucuronoxylomannan (GXM) and galactoxylomannan (GalXM) that is a well–established antiphagocytic factor (Kozel and Gotschlich, 1982, Zaragoza et al., 2009). It has been shown that *in vivo* and *in vitro* that phagocytosis of *C. neoformans* is dependent on opsonisation (Diamond, 1993, Levitz et al., 1999, Kozel, 1993, Zaragoza et al., 2003, Zaragoza et al., 2009, Leopold Wager et al., 2016) and that the capsule affects phagosome maturation (Smith et al., 2015). We showed that an acapsular mutant of *C. neoformans* was not significantly better phagocytosed than the wild-type strain. This is in accord with reports showing that *C. neoformans* exhibits capsule–independent phagocytosis–inhibitory mechanisms (García-Rodas and Zaragoza, 2012, Johnston and May, 2013) which are relevant in non–opsonic conditions, such as the induction of the expression of *GAT204* and *BLP1* (Chun et al., 2011, Smith et al., 2015).

For each fungal species, cell wall architecture changes as a result of morphological transitions and in response to predation by phagocytes and new environmental cues (Erwig and Gow, 2016, Wheeler et al., 2008, Ene et al., 2012, Hopke et al., 2016, Ballou et al., 2016, Pradhan et al., 2018, Pradhan et al., 2019, Briard et al., 2021). *S. cerevisiae, C. albicans* and *C. glabrata* have increased exposure of chitin and β-glucan at sites of budding), while *A. fumigatus* conidial swelling disrupts the poorly immunogenic rodlet layer and exposes melanin and the highly immunogenic β-glucan ligand (Aimanianda et al., 2009, Stappers et al., 2018) that is recognized by dectin-1 (Brown, 2006, Mentrup et al., 2022). Morphological changes can also result in the expression and exposure of a different set of immune ligands of the fungal surface. Unlike spores, *A. fumigatus* hyphae do not have rodlet and melanin layers in the outer cell wall but have α–1,3–glucan, galactomannan and galactosaminoglycan (Aimanianda et al., 2009, Garcia-Rubio et al., 2020). *C. albicans* hyphae express distinct cell–wall specific proteins, which are major antigens and which may act as invasins (Gow and Hube, 2012). Live cell imaging revealed how changes in fungal morphology correlated with macrophage uptake dynamics (McKenzie et al., 2010, Bain et al., 2021). The most significant changes in uptake occurred in cells undergoing morphogenesis, and these transitions are likely to significantly influence macrophage recognition. For example, resting *M. circinelloides* spores are prolate ellipsoids, while swollen spores are spherical. Higher uptake of swollen spores could reflect phagocytes preference for internalizing spherical shaped targets (Paul et al., 2013). Our earlier work also demonstrated that the orientation of *C. albicans* hyphae also influenced engulfment rate (Lewis et al., 2012).

Differential rates of phagosome acidification were observed following phagocytosis of the assessed fungi. Phagosomes containing the sporulating fungi *A. fumigatus* and *M. circinelloides* took longer to acidify. This observation supports other reports that showed that *A. fumigatus* is able to strongly inhibit phagosome acidification, which might relate to the presence of DHN–melanin and its biosynthetic precursors (Thywiβen et al., 2011). Previous reports have shown inhibition of phagosomal acidification by engulfed *M. circinellioides* spores (but not yeasts) (Lee et al., 2015, Pérez-Arques et al., 2019). Cell wall analysis of other zygomycetes showed that these fungi also produce DHN–melanin (Liu et al., 2021).

Heat killing fungal cells causes changes in cell wall architecture, such as increased exposure of β–glucan and mannan (Gow et al., 2007, Yadav et al., 2020). The uptake of heat-killed *C. albicans* and *A. fumigatus* was, however, reduced compared to live cells. Therefore, morphological transitions, such as *C. albicans* filamentation and *A. fumigatus* swelling, are likely to influence the efficiency of macrophage recognition. Morphogenesis not only modifies cell wall architecture and exposure of pathogen–associated molecular patterns (as discussed above), but can also lead to changes in the secretion and shedding of molecules that affect phagocyte–fungus interactions (Erwig and Gow, 2016). In contrast to this, heat–killing had no measurable effect upon the uptake of *C. neoformans cap*59Δ and *M. circinelloides*.

Previous studies have shown that complement opsonisation of *C. albicans* and *C. glabrata* differentially affect the fungus–phagocyte interaction (Singh et al., 2020). In this study we explored the effect of antibody opsonisation on the phagocytosis dynamics of these two *Candida* species. As shown previously (Rudkin et al., 2018), uptake of opsonized *C. albicans* with AB119 or AB140 by macrophages was faster than for non–opsonised cells. However, pre–incubation of *C. glabrata* with AB119 or AB140 did not affect uptake dynamics. It is likely that AB119 and AB140 bind to an epitope differentially expressed in *C. albicans* and *C. glabrata*, as indicated by immunofluorescence staining patterns.

This study therefore demonstrates the impact of cell wall structure, morphogenesis, and opsonisation upon macrophage phagocytosis dynamics and underlines the fact that differing cell wall composition markedly affects the timeframe to execute critical events of the phagocytic cycle. Furthermore, this study highlights the differential ability of innate immune cells to recognise, phagocytose and clear major fungal pathogens from divergent taxonomic families.

## CRediT authorship contribution statement

**María Fernanda Alonso:** Conceptualization, Data curation, Formal analysis, Investigation, Methodology, Validation, Visualization, Writing – original draft, Writing – review & editing. **Judith M. Bain:** Investigation, Methodology, Writing – review & editing. **Fiona M. Rudkin:** Investigation, Methodology, Writing – review & editing. **Lars P. Erwig:** Conceptualization, Writing – review & editing. **Alistair J.P. Brown:** Funding acquisition, Conceptualization, Writing – review & editing. **Neil A.R. Gow:** Conceptualization, Funding acquisition, Project administration, Resources, Validation, Visualization, Writing – original draft, Writing – review & editing.

## Declaration of Competing Interest

The authors declare the following financial interests/personal relationships which may be considered as potential competing interests: On behalf of the autrhors María Fernanda Alonso, Judith M. Bain, Fiona M. Rudkin, Lars P. Erwig, Alistair J.P. Brown, Neil A.R. Gow.
